# Supplementary material for: The open field assay is influenced by room temperature and by drugs that affect core body temperature
Source: F1000Res. 2024 Jul 3;12:234. Originally published 2023 Mar 2. [Version 4] doi: 10.12688/f1000research.130474.4 (PMC11165296; doi:10.12688/f1000research.130474.4)
Supplement: Peer review history

# Reviewer report: Reviewer response for version 4

**DOI:** 10.5256/f1000research.168314.r298857

**Published:** 2024-07-19

**Authors:** Spyridon Sideromenos

**Affiliations:** 1 Department of Neurophysiology and Neuropharmacology, Medical University of Vienna, Vienna, Austria

The authors' responses covered most of my main concerns and reservations, and, hence, I would like to approve the article.

Is the work clearly and accurately presented and does it cite the current literature?

Yes

If applicable, is the statistical analysis and its interpretation appropriate?

Yes

Are all the source data underlying the results available to ensure full reproducibility?

Yes

Is the study design appropriate and is the work technically sound?

Yes

Are the conclusions drawn adequately supported by the results?

Yes

Are sufficient details of methods and analysis provided to allow replication by others?

Yes

Reviewer Expertise:

NA

I confirm that I have read this submission and believe that I have an appropriate level of expertise to confirm that it is of an acceptable scientific standard.

# Reviewer report: Reviewer response for version 4

**DOI:** 10.5256/f1000research.168314.r298856

**Published:** 2024-07-10

**Authors:** Fiona Hollis

**Affiliations:** 1 Department of Pharmacology, Physiology and Neuroscience, University of South Carolina School of Medicine, Columbia, SC, USA

The authors have addressed my concerns by including limitations and performing additional statistical analyses (figure 3).

Is the work clearly and accurately presented and does it cite the current literature?

Partly

If applicable, is the statistical analysis and its interpretation appropriate?

Partly

Are all the source data underlying the results available to ensure full reproducibility?

Yes

Is the study design appropriate and is the work technically sound?

No

Are the conclusions drawn adequately supported by the results?

Partly

Are sufficient details of methods and analysis provided to allow replication by others?

No

Reviewer Expertise:

Behavioral neuroscience

I confirm that I have read this submission and believe that I have an appropriate level of expertise to confirm that it is of an acceptable scientific standard.

# Reviewer report: Reviewer response for version 3

**DOI:** 10.5256/f1000research.162395.r274455

**Published:** 2024-06-10

**Authors:** Kei Nagashima

**Affiliations:** 1 Body Temperature and Fluid Laboratory, Graduate School of Human Sciences, Faculty of Human Sciences, Waseda University, Saitama, Japan

I think that the present Manuscript has already been evaluated and revised properly to the comments. Therefore, I have no major concerns for this MS. However, I have some minor concerns for the use of terms.

 In abstract, icilin has agonistic action to TRBM8, but no thermolytic (cooling) action. What were controls for icilin. Ambient temperature of 23˚C is not thermoneutral condition for C57BL mice. The authors need to show more rationale for the selection of the temperature. In addition, 4˚C exposure is a cold stress, but may have induced pain sensation. The subsequent behavioral response may not have been related to thermoregulatory responses and behavior.

Is the work clearly and accurately presented and does it cite the current literature?

Yes

If applicable, is the statistical analysis and its interpretation appropriate?

Yes

Are all the source data underlying the results available to ensure full reproducibility?

Yes

Is the study design appropriate and is the work technically sound?

Yes

Are the conclusions drawn adequately supported by the results?

Yes

Are sufficient details of methods and analysis provided to allow replication by others?

Yes

Reviewer Expertise:

Environmental physiology, Thermoregulation

I confirm that I have read this submission and believe that I have an appropriate level of expertise to confirm that it is of an acceptable scientific standard.

# Reviewer report: Reviewer response for version 3

**DOI:** 10.5256/f1000research.162395.r274454

**Published:** 2024-06-05

**Authors:** Spyridon Sideromenos

**Affiliations:** 1 Department of Neurophysiology and Neuropharmacology, Medical University of Vienna, Vienna, Austria

In this study, by Jimenez et.al, the effects of an ambient cold temperature (4°C) in the open field are described. In addition, the authors  pharmacologically inhibited or activated the cold-sensing TRPM-8 cation receptors, in mice exposed at cold ambient temperature (4°C), and described the interactions between TRPM-8 channel and cold exposure, as observed in the open field test.

 While is quite an interesting, I would like to encourage the authors to address the following comments:

 1) In the main text the open field test lasted for 1 hours. However in the abstract it is mentioned 'Mice were placed in the open field at 4°C and 23°C for 30 minutes'.

 2) Did the authors use male or female mice? And why? It should be always clearly stated.

 3) Can the authors explain the rationale for the doses tested for all compounds, as well as include references with the effects on these compounds in the open field test?

 4) The authors are  rather measuring the skin temperature and not core body temperature. Can they please explain? They should also discuss how skin temperature correlates with CBT. There are also drugs that can solely influence the skin temperature. It should be noted.

 5) In figure 2, Icilin alter skin temperature  90 mins post-injection. However, the open field starts 10 min after the injection and lasts for 60 mins (i.e. 70 mins post-injection).

 How, then, they concluded that altered temperature influences the open field behavior. Can they please explain?

 6) In figure 6, it should be noted that no vehicle-injected mice were used. It is common that skin temperature increases and decreases rapidly upon i.p injections. Without an appropriate control experiments, it is rather difficult to confidently conclude whether diazepam, indeed, reduces body temperature. That is certainly a limitation and should be noted.

 7) Its extremely unclear what is the difference of figure 6 and 3. In this graph it seems that  diazepam has no effect at all.

 The authors should explain and also further discuss these results in conclusion.

Is the work clearly and accurately presented and does it cite the current literature?

Yes

If applicable, is the statistical analysis and its interpretation appropriate?

Yes

Are all the source data underlying the results available to ensure full reproducibility?

Yes

Is the study design appropriate and is the work technically sound?

Yes

Are the conclusions drawn adequately supported by the results?

Yes

Are sufficient details of methods and analysis provided to allow replication by others?

Yes

Reviewer Expertise:

Neuroscience

I confirm that I have read this submission and believe that I have an appropriate level of expertise to confirm that it is of an acceptable scientific standard, however I have significant reservations, as outlined above.

# Response

**Published:** 2024-06-26

**Authors:** Mark Zylka

**Affiliations:** The University of North Carolina at Chapel Hill, USA

In this study, by Jimenez et.al, the effects of an ambient cold temperature (4°C) in the open field are described. In addition, the authors pharmacologically inhibited or activated the cold-sensing TRPM-8 cation receptors, in mice exposed at cold ambient temperature (4°C), and described the interactions between TRPM-8 channel and cold exposure, as observed in the open field test. While is quite an interesting, I would like to encourage the authors to address the following comments:

 1) In the main text the open field test lasted for 1 hours. However in the abstract it is mentioned 'Mice were placed in the open field at 4°C and 23°C for 30 minutes'.

**Ans: **The open field was run for 1 hour. The abstract method was corrected.

 2) Did the authors use male or female mice? And why? It should be always clearly stated.

**Ans: **As stated in the Abstract Background, male WT and TRPM8 knock-out mice were used. This is now restated in the methods and discussion as a limitation. “For consistency, only male mice were used in this study. However, it should be stated that female mice may display different drug-induced behavioral responses and should be explored in future studies.”

 3) Can the authors explain the rationale for the doses tested for all compounds, as well as include references with the effects on these compounds in the open field test?

**Ans: **This comment was addressed in the previous revision with appropriate reference “Here we selected drug doses that are commonly used and well tolerated by mice in the behavior field.”

 Almeida et al. used 6 mg/kg i.p. M8-B to evaluate the hypothermic action of the drug. We used 12 mg/kg as we didn’t see an effect with 6 mg/kg.

 Vinkers et al. evaluated stress-induced hyperthermia following 1-4 mg/kg diazepam. Padua-Reis et al. also evaluated the anxiolytic effect of 2 mg/kg diazepam. We used 2 mg/kg.

*New reference:* Colburn et al. evaluated icilin-induced jumping and cold sensation following 60 mg/kg icilin administration (new reference). We used 50 mg/kg.

 4) The authors are rather measuring the skin temperature and not core body temperature. Can they please explain? They should also discuss how skin temperature correlates with CBT. **Ans: **There are also drugs that can solely influence the skin temperature. It should be noted.

 We did not measure skin temperature. CBT was assessed by measuring rectal temperature in mice, a technique that has been widely used for many years. Limitations of this procedure include distress to the animals, which could increase core temperature. However, however this risk was reduced by acclimating the mice to the procedure 2x each day for one week prior to testing. This was added to the methods.  

 5) In figure 2, Icilin alter skin temperature  90 mins post-injection. However, the open field starts 10 min after the injection and lasts for 60 mins (i.e. 70 mins post-injection). How, then, they concluded that altered temperature influences the open field behavior. Can they please explain?

**Ans: **Previous studies had already shown a rapid change in core body temperature post-icilin injection using thermal telemetry and at doses lower than the one used in the current study, and these significant changes in CBT lasted at least 90 minutes (Knowlton et al, 2011 – *New Reference*). Using a less sensitive device, we did see an increase in CBT during the open field studies consistent with these studies. We likely did not see a change in CBT earlier due to our low sample size and less sensitive temperature detection method. This information was added to the results section.

 6) In figure 6, it should be noted that no vehicle-injected mice were used. It is common that skin temperature increases and decreases rapidly upon i.p injections. Without an appropriate control experiments, it is rather difficult to confidently conclude whether diazepam, indeed, reduces body temperature. That is certainly a limitation and should be noted.

**Ans: **To consider temperature changes upon i.p. injections, the results from figures 4 and 6 are compared with their saline-injected vehicle controls in figure 5. Diazepam was dissolved in saline and M8-B was dissolved DMSO. This is included in the methods section. The following limitation is included in the discussion as a previous revision: “Of note, our vehicle controls in Figure 5 were saline injected, which may limit the interpretation of this study.” However, “DMSO at the concentration used to dissolve Icilin and M8-B have also been reported to be well tolerated by mice”.  

 7) Its extremely unclear what is the difference of figure 6 and 3. In this graph it seems that  diazepam has no effect at all. The authors should explain and also further discuss these results in conclusion.

**Ans: **Figure 6 represents the effect of diazepam on CBT and open field behavior in WT and Trpm8 knock out mice. Panel A shows the effect of diazepam on the core body temperature. Panel B shows the effect of diazepam on the open field behavior of these two genotypes at 4C.

 Figure 3 represents the effect of temperature on the open field behavior of WT and Trpm8 knock out mice. Mice are not injected with drugs in this experiment. Both genotypes are assessed in the open field at room temperature and in the cold room at 4C. A new graph was included in the previous revision to better represent this experiment.

# Reviewer report: Reviewer response for version 2

**DOI:** 10.5256/f1000research.159192.r228500

**Published:** 2024-01-23

**Authors:** Fiona Hollis

**Affiliations:** 1 Department of Pharmacology, Physiology and Neuroscience, University of South Carolina School of Medicine, Columbia, SC, USA

I thank the authors for addressing many of my concerns. Please note that the figure legend for Figure 4A-C is still mislabeled. I believe that the temperature panel A was added after the legend was written (as the legend is shifted and missing panel C). The authors might also consider adding an additional limitation to their discussion that the lighting in the open field is different between their temperature conditions. Ideally, the lighting intensity would be the same since the conditions are being directly compared, however, as both lighting conditions are towards the high end, and the condition with lower lighting has animals exhibiting higher levels of avoidance, it is likely not a significant factor. I still have reservations about the lack of appropriate vehicle controls. The authors have added a statement in the discussion stating that DMSO is used at a dose that is well-tolerated, but I feel that they should state the limitation that the effects of DMSO are not controlled for in these experiments. Pending these revisions, the report will be satisfactory.

Is the work clearly and accurately presented and does it cite the current literature?

Partly

If applicable, is the statistical analysis and its interpretation appropriate?

Partly

Are all the source data underlying the results available to ensure full reproducibility?

Yes

Is the study design appropriate and is the work technically sound?

No

Are the conclusions drawn adequately supported by the results?

Partly

Are sufficient details of methods and analysis provided to allow replication by others?

No

Reviewer Expertise:

Behavioral neuroscience

I confirm that I have read this submission and believe that I have an appropriate level of expertise to confirm that it is of an acceptable scientific standard, however I have significant reservations, as outlined above.

# Reviewer report: Reviewer response for version 1

**DOI:** 10.5256/f1000research.143236.r165350

**Published:** 2023-10-13

**Authors:** Fiona Hollis

**Affiliations:** 1 Department of Pharmacology, Physiology and Neuroscience, University of South Carolina School of Medicine, Columbia, SC, USA

The authors have examined Open Field behavior in different temperatures to better understand the effects of temperature fluctuations on behavior. This is an important topic that should be better explored. Here, the experiments, as described, do not appear rigorous enough to support the conclusions. Moreover, the discussion does not situate the manuscript within the wider context of the literature, nor discuss the limitations or alternative interpretations of these experiments. Main concerns are detailed below:

 The authors show temperature variations that are within 2-4C between seasons/rooms/buildings. However, experiments were conducted at vastly different temperatures (4C and 23C) and the conclusion drawn that “it is important to consider temperature fluctuations”. The authors do not show such wild temperature fluctuations in Figure 1, so what is the rationale for examining behavior at 4C – a temperature that nearly every laboratory building will not be near (except cold rooms)? A more logical experiment (particularly in light of the authors’ temperature-controlled environment) would be to measure behavior at the differing temperatures that they observe across seasons or buildings. This would better reflect the variation in the literature between universities and provide insight into conflicting data. If there was no effect of those temperatures on behavior, then it could be concluded that fluctuations within 2-4C do not affect this particular behavior.

 Can the authors please discuss the rationale for their drug doses. The diazepam dose in particular has been found to be anxiogenic and potentially sedative in mice ^1^.

 Peripheral DMSO injections can be toxic to mice above 10%. The methods suggest that drugs were dissolved in pure DMSO and then injected i.p. Can the authors please verify that the DMSO levels were not at toxic or noxious levels? Peripheral DMSO has been shown to be painful and if drugs are administered in pure DMSO, then behavioral interpretations may be limited.

 Open Field behavior is highly influenced by lighting conditions. The authors should indicate the lighting conditions of the test in lux.

 In Figure 3, the authors compare the effects of temperature on open field, but present the data in separate graphs. Are the same animals tested at both temperatures? If so, the data should be analyzed by repeated measures ANOVA and the temperature considered as an additional factor to genotype. This will allow the authors to make the claim of more or less effects of cold temperature on OF behavior.

 In Figure 4, are these the same groups of animals as in Figure 3? The authors should specify whether each experiment used naïve animals or those from previous experiments. The authors injected a TRPM8 antagonist but do not include vehicle-injected groups. As this drug is diluted in DMSO, this group is necessary. Moreover, the authors are making comparisons to non-injected animals in Figure 3. Statistically and experimentally, that is inappropriate unless Figure 3 animals were injected with vehicle (in which case, this needs to be properly stated and the statistics performed on all groups).

 Figure 5, saline is used as the vehicle for diazepam, but again, there is no vehicle for M8-B (which the authors state is diluted in DMSO). This is inappropriate as DMSO injections evoke different reactions than saline injections. The results state that the reduction in CBT was associated with a near-significant increase in OF behavior in WT but not mutant mice, however, there are no correlations shown between behavior and temperature (did behavior change across time as CBT decreased?). Moreover, in this case, the OF was performed 30min after Diazepam administration for 1h, per the methods. Was CBT measured during the OF then? How did the authors confirm that behavior was not disrupted by this measurement? Was CBT measured at a separate time point from behavior? In which case, were the animals naïve or repeatedly injected? Finally, diazepam appears to have no effect on behavior at 4C – the authors should verify that their dose is working well at 23C to ensure that there are no confounding sedative or anxiogenic effects.

 The discussion is incredibly brief and does not do anything to discuss potential limitations, explanations, or situate the manuscript within the literature. There is mention of a single review. There are other publications that discuss the pitfalls of temperature fluctuations that should be included here. The authors need to also consider the literature of Trpm8’s function and role.

 Minor: Figure 4 figure legend is mislabeled.

Is the work clearly and accurately presented and does it cite the current literature?

Partly

If applicable, is the statistical analysis and its interpretation appropriate?

Partly

Are all the source data underlying the results available to ensure full reproducibility?

Yes

Is the study design appropriate and is the work technically sound?

No

Are the conclusions drawn adequately supported by the results?

Partly

Are sufficient details of methods and analysis provided to allow replication by others?

No

Reviewer Expertise:

Behavioral neuroscience

I confirm that I have read this submission and believe that I have an appropriate level of expertise to state that I do not consider it to be of an acceptable scientific standard, for reasons outlined above.

## References

[1] : Diazepam causes sedative rather than anxiolytic effects in C57BL/6J mice. *Sci Rep* .2021;11(1) : 10.1038/s41598-021-88599-5 9335 10.1038/s41598-021-88599-5 33927265 PMC8085115

# Response

**Published:** 2023-11-20

**Authors:** Mark Zylka

**Affiliations:** The University of North Carolina at Chapel Hill, USA

The authors have examined Open Field behavior in different temperatures to better understand the effects of temperature fluctuations on behavior. This is an important topic that should be better explored. Here, the experiments, as described, do not appear rigorous enough to support the conclusions. Moreover, the discussion does not situate the manuscript within the wider context of the literature, nor discuss the limitations or alternative interpretations of these experiments. Main concerns are detailed below:

 The authors show temperature variations that are within 2-4C between seasons/rooms/buildings. However, experiments were conducted at vastly different temperatures (4C and 23C) and the conclusion drawn that “it is important to consider temperature fluctuations”. The authors do not show such wild temperature fluctuations in Figure 1, so what is the rationale for examining behavior at 4C – a temperature that nearly every laboratory building will not be near (except cold rooms)? A more logical experiment (particularly in light of the authors’ temperature-controlled environment) would be to measure behavior at the differing temperatures that they observe across seasons or buildings. This would better reflect the variation in the literature between universities and provide insight into conflicting data. If there was no effect of those temperatures on behavior, then it could be concluded that fluctuations within 2-4C do not affect this particular behavior.

**>While the testing environments used in this study (23C and 4C) represent a wider temperature range than is observed between laboratories, this proof of principle study demonstrates that temperature can have an influence on behavior. More importantly, in this study we demonstrate that the body temperature changes induced following drug administration could lead to similar physiological responses induced by normal room temperature fluctuations. We have included in the discussion this limitation in interpretation *.***

 Can the authors please discuss the rationale for their drug doses. The diazepam dose in particular has been found to be anxiogenic and potentially sedative in mice ^1^ .

**>The drug doses were chosen based on those commonly used in the literature to study open field behavior. While we cannot eliminate the influence the drugs have on the entire body, the end goal was to make an association between behavior and body temperature. This limitation has been included in the discussion.**

 Peripheral DMSO injections can be toxic to mice above 10%. The methods suggest that drugs were dissolved in pure DMSO and then injected i.p. Can the authors please verify that the DMSO levels were not at toxic or noxious levels? Peripheral DMSO has been shown to be painful and if drugs are administered in pure DMSO, then behavioral interpretations may be limited.

**>DMSO did not exceed 2mL/kg. Studies show that mice can tolerate 10mL/kg. This limitation has been included in the discussion.**

 (Gad SC, Cassidy CD, Aubert N, Spainhour B, Robbe H. Nonclinical Vehicle Use in Studies by Multiple Routes in Multiple Species. International Journal of Toxicology. 2006;25(6):499-521.)

 Open Field behavior is highly influenced by lighting conditions. The authors should indicate the lighting conditions of the test in lux.

**>The lighting conditions in the room temperature and cold room studies were measured at 660 lux and 400 lux, respectively. This measurement has been added to the methods section.**

 In Figure 3, the authors compare the effects of temperature on open field, but present the data in separate graphs. Are the same animals tested at both temperatures? If so, the data should be analyzed by repeated measures ANOVA and the temperature considered as an additional factor to genotype. This will allow the authors to make the claim of more or less effects of cold temperature on OF behavior.

**>Yes the same animals were tested in both temperatures. The data has been analyzed using a mixed effects analysis with multiple comparisons, Sidak. Figure 3 has been updated.**

 In Figure 4, are these the same groups of animals as in Figure 3? The authors should specify whether each experiment used naïve animals or those from previous experiments. The authors injected a TRPM8 antagonist but do not include vehicle-injected groups. As this drug is diluted in DMSO, this group is necessary. Moreover, the authors are making comparisons to non-injected animals in Figure 3. Statistically and experimentally, that is inappropriate unless Figure 3 animals were injected with vehicle (in which case, this needs to be properly stated and the statistics performed on all groups).

**>Yes, the same animals are used throughout the study except for Figure 2. The tests were conducted with a 1 week recovery period in between. This statement is included in the methods. The vehicle saline injected group is included in figure 5 for comparison. Comparison was not made to DMSO injected mice. This limitation is included in the discussion.**

 Figure 5, saline is used as the vehicle for diazepam, but again, there is no vehicle for M8-B (which the authors state is diluted in DMSO). This is inappropriate as DMSO injections evoke different reactions than saline injections. The results state that the reduction in CBT was associated with a near-significant increase in OF behavior in WT but not mutant mice, however, there are no correlations shown between behavior and temperature (did behavior change across time as CBT decreased?). Moreover, in this case, the OF was performed 30min after Diazepam administration for 1h, per the methods. Was CBT measured during the OF then? How did the authors confirm that behavior was not disrupted by this measurement? Was CBT measured at a separate time point from behavior? In which case, were the animals naïve or repeatedly injected? Finally, diazepam appears to have no effect on behavior at 4C – the authors should verify that their dose is working well at 23C to ensure that there are no confounding sedative or anxiogenic effects.

**>The rectal temperatures were not measured during the same time as the open field tests. The temperature and behavior tests were conducted one week apart. DMSO injected mice were not included in the study. This limitation has been included in the discussion.**

 The discussion is incredibly brief and does not do anything to discuss potential limitations, explanations, or situate the manuscript within the literature. There is mention of a single review. There are other publications that discuss the pitfalls of temperature fluctuations that should be included here. The authors need to also consider the literature of Trpm8’s function and role.

 Minor: Figure 4 figure legend is mislabeled.

# Response

**Published:** 2024-02-06

**Authors:** Mark Zylka

**Affiliations:** The University of North Carolina at Chapel Hill, USA

We addressed minor comments regarding interpretation of results and limitations in the Discussion section.
